# Metagenomic Analysis Identifies Sex‐Related Gut Microbial Functions and Bacterial Taxa Associated With Skeletal Muscle Mass

**DOI:** 10.1002/jcsm.13636

**Published:** 2024-11-19

**Authors:** Hang A. Park, Joohon Sung, Yoosoo Chang, Seungho Ryu, Kyung Jae Yoon, Hyung‐Lae Kim, Han‐Na Kim

**Affiliations:** ^1^ Genome and Health Big Data Laboratory, Graduate School of Public Health Seoul National University Seoul Republic of Korea; ^2^ Department of Emergency Medicine, Hallym University Dongtan Sacred Heart Hospital Gyeonggi‐do Republic of Korea; ^3^ Institute of Health and Environment Seoul National University Seoul Republic of Korea; ^4^ Genomic Medicine Institute Seoul National University Seoul Republic of Korea; ^5^ Center for Cohort Studies, Total Healthcare Center, Kangbuk Samsung Hospital Sungkyunkwan University School of Medicine Seoul Republic of Korea; ^6^ Department of Occupational and Environmental Medicine, Kangbuk Samsung Hospital Sungkyunkwan University School of Medicine Seoul Republic of Korea; ^7^ Department of Clinical Research Design and Evaluation, Samsung Advanced Institute for Health Sciences and Technology Sungkyunkwan University Seoul Republic of Korea; ^8^ Department of Physical and Rehabilitation Medicine, Kangbuk Samsung Hospital Sungkyunkwan University School of Medicine Seoul Republic of Korea; ^9^ Department of Biochemistry, College of Medicine Ewha Womans University Seoul Republic of Korea; ^10^ Biomedical Statistics Center, Research Institute for Future Medicine Samsung Medical Center Seoul Republic of Korea

**Keywords:** gut microbiome, metagenomics, microbial function, sarcopenia, skeletal muscle mass

## Abstract

**Background:**

This study aimed to explore the association between gut microbiota functional profiles and skeletal muscle mass, focusing on sex‐specific differences in a population under 65 years of age.

**Methods:**

Stool samples from participants were analysed using metagenomic shotgun sequencing. Skeletal muscle mass and skeletal muscle mass index (SMI) were quantified (SMI [%] = total appendage muscle mass [kg]/body weight [kg] × 100) using bioelectrical impedance analysis. Participants were categorized into SMI quartiles, and associations between gut microbiota, functional profiling and SMI were assessed by sex, adjusting for age, BMI and physical activity.

**Results:**

The cohort included 1027 participants (651 men, 376 women). In men, *Escherichia coli* (log2 fold change 3.08, *q* = 0.001), *Ruminococcus_B gnavus* (log2 fold change 2.89, *q* = 0.014) and *Enterocloster sp001517625* (log2 fold change 2.47, *q* = 0.026) were more abundant in the lowest SMI compared to the highest SMI group. In contrast, *Bifidobacterium bifidum* (log2 fold change 3.13, *q* = 0.025) showed higher levels in the second lowest SMI group in women. Microbial pathways associated with amino acid synthesis (MET‐SAM‐PWY: log2 fold change 0.42; METSYN‐PWY: log2 fold change 0.44; SER‐GLYSYN‐PWY: log2 fold change 0.20; PWY‐5347: log2 fold change 0.41; P4‐PWY: log2 fold change 0.53), *N*‐acetylneuraminate degradation (log2 fold change 0.43), isoprene biosynthesis (log2 fold change 0.20) and purine nucleotide degradation and salvage (PWY‐6353: log2 fold change 0.42; PWY‐6608: log2 fold change 0.38; PWY66‐409: log2 fold change 0.52; SALVADEHYPOX‐PWY: log2 fold change 0.43) were enriched in the lowest SMI in men (*q* < 0.10). In women, the second lowest SMI group showed enrichment in energy‐related pathways, including lactic acid fermentation (ANAEROFRUCAT‐PWY: log2 fold change 0.19), pentose phosphate pathway (PENTOSE‐P‐PWY: log2 fold change 0.30) and carbohydrate degradation (PWY‐5484: log2 fold change 0.31; GLYCOLYSIS: log2 fold change 0.29; PWY‐6901: log2 fold change 0.27) (*q* < 0.05).

**Conclusions:**

This study highlights sex‐specific differences in gut microbiota and functional pathways associated with SMI. These findings suggest that gut microbiota may play a role in muscle health and point toward microbiota‐targeted strategies for maintaining muscle mass.

## 1 Introduction

Skeletal muscle plays a vital role in daily activities, and its age‐related decline leads to weakness and functional limitations [1]. Sarcopenia, characterized by the loss of skeletal muscle mass and function, poses a serious public health burden, contributing to physical disabilities and increased all‐cause mortality rates [2]. In 2019, the annual cost of hospitalization for one person with sarcopenia was reportedly over $2315 in the United States and £2707 in the United Kingdom [3, 4]. Estimates also suggest that reducing the prevalence of sarcopenia by 10% would reduce US healthcare costs by $1.1 billion per year [5]. Therefore, identifying individuals at risk of skeletal muscle loss and understanding the underlying mechanisms of skeletal muscle wasting are important.

Sex‐specific mechanisms may play a crucial role in muscle loss. Previous studies have reported sex differences in the prevalence and symptoms of sarcopenia, with higher prevalence and faster age‐dependent muscle mass loss observed in men than women [6, 7]. RNA sequence‐based gene expression analysis of vastus lateralis muscle biopsies also revealed that gene expression is regulated in the same direction in both sexes during muscle aging, but there were sex‐specific differences in the magnitude of the changes [8]. These differences may stem from sex‐dependent mechanisms associated with skeletal muscle loss. Despite these studies identifying sex‐specific markers of muscle loss, data in this area remain limited.

The emerging concept of the ‘gut‐muscle axis’ refers to the bidirectional relationship between the gut microbiota and skeletal muscle [9]. This concept suggests that the composition and functionality of gut microbiota can influence muscle health, such as the metabolic function and quality of muscle tissue [10], while muscle activity and health can, in turn, impact gut microbiota. The gut–muscle axis has also been identified as a potential biological target for preventing and treating muscle‐related diseases, such as sarcopenia and muscular dystrophy [11]. However, the complex mechanisms underlying this axis are not yet fully understood, highlighting the need for further research into gut microbial function.

Microbial composition differs by sex, likely influenced by sex hormones [12, 13], and these differences may contribute to variations in physiological traits, including muscle mass. In our previous study using 16S rRNA gene sequencing, we observed notable sex differences in the correlation between gut microbiota and skeletal muscle index (SMI) [14]. This finding highlighted the potential impact of sex on the gut–muscle axis and motivated us to explore the underlying mechanisms in detail. Investigating the association between gut microbiota and sex‐specific skeletal muscle mass could offer valuable insights into the gut–muscle axis.

While there is evidence of the gut microbiome's influence on muscle mass, sex‐specific differences in microbial function remain poorly understood. Most research has focused on microbial composition, but understanding the functional roles of these microbes is essential for identifying therapeutic targets. Functional profiling can reveal metabolic pathways and biological functions, offering insights into host–microbe interactions. Given the recognized disparities in muscle loss between sexes, this study investigates the association between gut microbiome profiles—both taxonomic and functional—and skeletal muscle mass by sex in a large population‐based cohort, using metagenomic shotgun sequencing to explore these differences.

## 2 Methods

### 2.1 Study Design and Subjects

**FIGURE 1:**
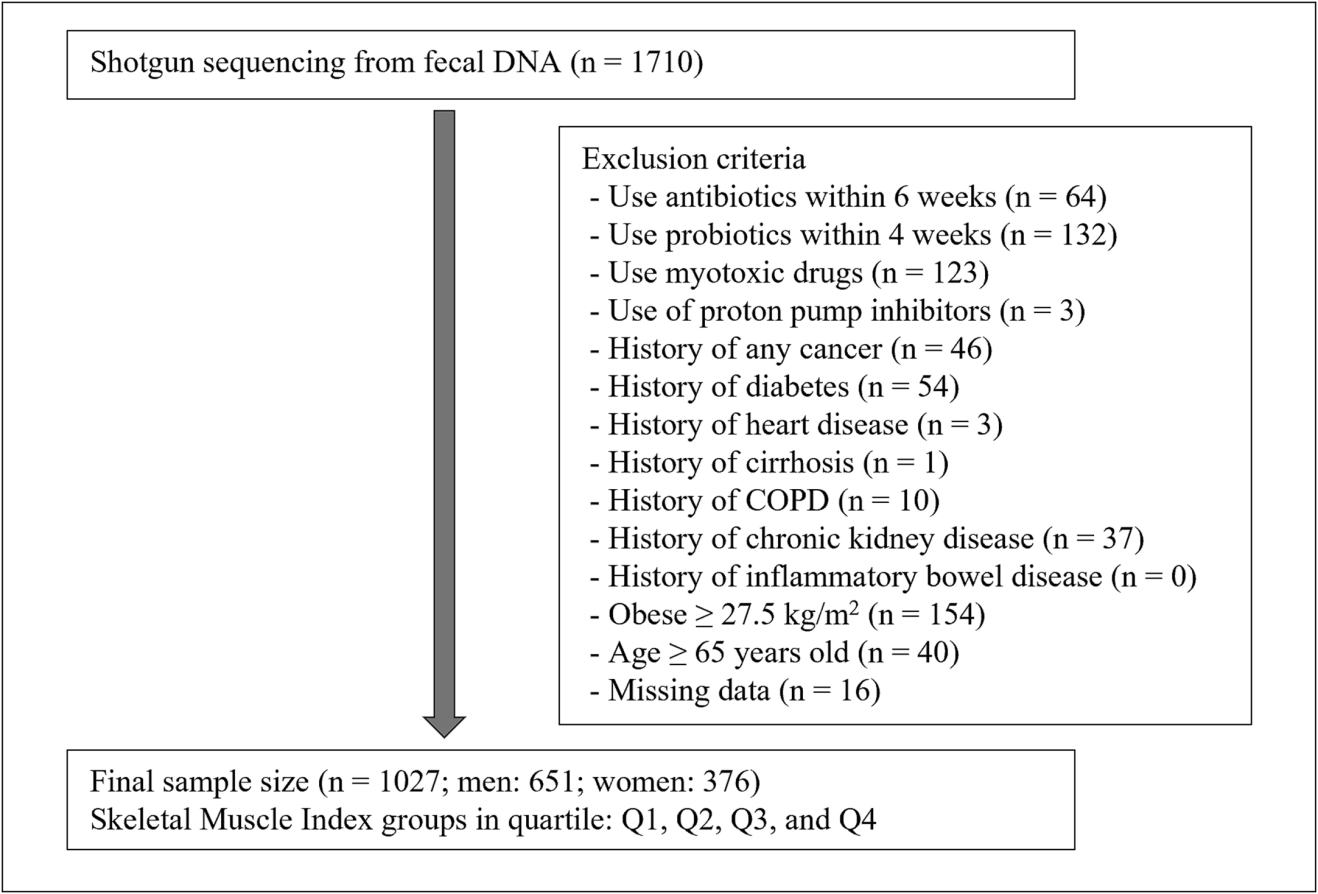
Study flow. COPD, chronic obstructive pulmonary disease.

We recruited Korean men and women aged 25–78 years who underwent comprehensive annual or biennial physical examinations as part of workplace health check‐ups between June 2014 and May 2021 at the Kangbuk Samsung Hospital Healthcare Screening Center in the Republic of Korea. Fecal samples were collected from participants aged 25–64 years. Participants were excluded based on the following criteria: use of antibiotics within 6 weeks prior to enrolment, use of probiotics within 4 weeks prior to enrolment, current use of myotoxic medications or proton pump inhibitors, and a history of any cancer, chronic obstructive pulmonary disease, cirrhosis, diabetes, heart disease, chronic kidney disease or inflammatory bowel disease. Additionally, participants with a body mass index (BMI) of 27.5 kg/m^2^ or higher (as per the World Health Organization Asia BMI cut‐off), those aged 65 years or older, and those with missing variables were also excluded (Figure 1).

### 2.2 Data Collection and Variables

Participants completed self‐administered questionnaires, including a 103‐item self‐administered food frequency questionnaire developed for use in South Korea, to assess dietary intake [15]. Physical activity was assessed using the International Physical Activity Questionnaire–116 short form, which examined the number of days per week that participants engaged in vigorous physical activity for ≥ 20 min per session. Fasting insulin, glucose, triglyceride, and alanine aminotransferase (ALT) levels were measured in blood samples collected by a nurse in the morning from the antecubital vein of patients who had fasted for more than 12 hours. Body measurements included height, weight, skeletal muscle mass, and body fat mass.

A bioelectrical impedance analysis (BIA) device equipped with eight‐point tactile electrodes (InBody 720, Biospace Co., Seoul, Korea) was used to calculate skeletal muscle mass and body fat mass. Participants underwent BIA measurements while in a fasting state; they stood barefoot and grasped the handles of the analyser to ensure proper contact with the electrodes. The subjects were instructed to empty their bladders before measurement. All procedures were conducted according to the manufacturer's instructions. The BIA was calibrated and validated for the accuracy and reproducibility of the body composition index [16]. SMI was calculated using the following formula: SMI (%) = skeletal muscle mass (kg)/body weight (kg) × 100, adjusted for body weight according to previously established methods [17]. The participants were categorized into four groups based on their SMI quartiles: Q1 (lowest SMI group), Q2, Q3 and Q4 (highest SMI group).

### 2.3 DNA Extraction, Shotgun Metagenomic Sequencing and Quantity Control of Reads

Fecal samples were collected at participants' homes within 24 h prior to their hospital visit. Participants were provided in advance with the OMNIgene‐GUT collection kit (OMR‐200; DNA Genotek, Ottawa, Canada), a stick for collection and detailed instructions on how to collect and store feces. They were instructed to defecate onto a stool collection sheet placed above the toilet water, transfer a sample of the stool into the kit's container, which requires no preservative, and then immediately store the sample in a −20°C freezer. Upon arrival at the hospital, the samples were promptly transferred to a −70°C freezer in the laboratory. Microbial genomic DNA was extracted from the fecal samples within 1 month using the DNeasy PowerSoil Pro Kit (Qiagen, Hilden, Germany), following the manufacturer's instructions.

Paired‐end sequencing (2 × 150 bp) was carried out on Illumina Hiseq 4000 platform (2 × 150 bp) at Macrogen, Korea, generating a total of over 10.3 T bp (an average of 7.4G bp per sample) and 67.8G reads for 1710 samples. The shotgun metagenomic reads were preprocessed by removing adapter sequences, and low‐quality reads were preprocessed using Trimmomatic (ver. 0.39) [18]. Trimmomatic was run using the parameters SLIDING WINDOW:4:20 MINLEN:75. To remove host DNA sequences, all metagenomic reads were mapped against the human (hg38) and PhiX genomes using BWA (ver. 0.7.17) [19], and Picard (ver. 2.27.2) tools (http://broadinstitute.github.io/picard/) were used to obtain reads of non‐human DNA, producing a mean (SD) of 42.4 M (11.4 M) reads per sample (an average of 6.4G bp per sample) for the metagenomic analysis.

### 2.4 Taxonomic, Functional and Metabolic Profiling

Taxonomic profiling of the metagenomic samples was performed using MetaPhlAn 4.0 [20]. Default settings were used to generate taxonomic relative abundances. Microbial functional profiles were estimated using HUMAnN 3.0 (HMP Unified Metabolic Analysis Network) with the Chocophlan nucleotide and Uniref90 protein databases [21, 22]. The MetaCyc metabolic pathway database included in HUMAnN 3.0 was used to obtain the MetaCyc pathway abundances detected per sample [23]. To predict the metabolites in the microbiome, we used MelonnPan, which predicts the relative abundance of each metabolite in each sample using an elastic net linear regression model to predict the relative abundance of each metabolite in each sample [24].

### 2.5 Diversity Analysis

Alpha diversity was assessed by calculating the number of species observed in each sample, Shannon's index and Simpson's index, whereas beta diversity estimates included Bray–Curtis dissimilarity and Jaccard's index. To determine statistically significant differences in the alpha and beta diversity indices between the SMI groups, we used linear regression and permutation analysis of variance (PERMANOVA) with 999 random permutations using the *adonis2* function in the vegan R package, respectively [25]. The variable for the SMI group was included as the last term in the PERMANOVA formula. All *p*‐values were adjusted to account for multiple comparisons using Benjamini–Hochberg false discovery rate (FDR) correction, and an adjusted *p*‐value (*q*‐value) of < 0.05 was considered significant.

### 2.6 Statistical Analysis and Visualization

To determine the taxa that were differentially abundant between the SMI groups, we used a linear differential abundance analysis model (LinDA), which is based on a linear regression model for centred log‐ratio‐transformed data and corrects for bias due to composition effects [26]. Differentially abundant functional pathways and predicted metabolites according to SMI groups were also assessed using LinDA. The LinDA analysis used default parameters, including feature data types as proportions; the minimum abundance of each feature was set to 0.001 (0.1%), and the prevalence filter was set as follows: 0.05 (5%) for taxa and metabolites and 0.10 (10%) for pathways. All *p*‐values were also corrected for multiple testing using FDR (*q*‐values), and *q*‐values of < 0.10 were considered significant. Bar plots were used to visualize the major species contributing to the microbial functions identified in the differential analysis. We also examined the correlations between species that were differentially abundant between the SMI groups (*q <* 0.10) and pathways that were significantly associated with the SMI groups (*q < 0*.10). Heatmap representations of the correlations were generated using the R package ComplexHeatmap [27].

We conducted the analyses separately for men and women. The basic demographics of the study participants were compared according to the SMI group. Continuous variables with a normal distribution are presented as means (standard deviation [SD]), and non‐normally distributed variables are presented as medians (interquartile range [IQR]). Categorical variables are presented as counts and percentages. Continuous variables were compared using the *t‐*test or Mann–Whitney *U* test, while the frequencies of categorical variables were compared using the chi‐squared test. To examine linear trends with increasing SMI quartiles, linear regression or the Jonkheere–Terpstara (non‐parametric) test was used for continuous variables, and the Cochran–Armitage trend test was used for categorical variables.

All analyses were adjusted for age and BMI to account for the potential impact of confounders on the microbiome. For men, the number of days of vigorous physical activity per week was significantly different between the groups (*p* < 0.001) and was therefore included as a covariate in the analysis. However, there was no significant difference in the number of days of vigorous physical activity between the groups for women (*p* = 0.131), so it was not included as a covariate. Although smoking can also affect microbial composition, it was not used as a covariate in our study because there was no significant difference in smoking status between the groups for both sexes. Nutrient variables were similarly not included as covariates since they did not differ significantly between the groups.

### 2.7 Ethics Approval and Consent to Participate

The study protocol was conducted in accordance with the Declaration of Helsinki and approved by the Institutional Review Board (IRB) of Seoul National University (SNU 24‐04‐127). Written informed consent was obtained from all participants after explaining all the possible consequences of the study.

## 3 Results

### 3.1 Baseline Characteristics of Study Population by Sex

During the study period, 1710 individuals participated. After excluding participants based on the exclusion criteria, 1027 participants were included in the analysis: 651 men and 376 women (Figure 1). There were 778 participants included in the previous 16S rRNA gene analysis (456 men, 322 women) [14], and the remaining 249 subjects were additionally recruited. Baseline characteristics of the patients are presented in Table 1. The mean (SD) of skeletal muscle mass was higher in men (31.2 kg [3.3]) than in women (21.1 kg [2.4]). To observe sex‐specific trends, we divided the SMI group into quartiles for both men and women. We found that nutritional variables, including total calories, protein, fat, carbohydrates and fibre, did not exhibit significant trends across the SMI quartiles for either sex. In both men and women, higher SMI quartiles were associated with lower age and BMI, serum insulin, glucose, triglyceride and ALT levels. The number of days of vigorous physical activity per week showed no difference between groups in women but increased significantly with higher SMI quartiles in men.

**TABLE 1:** Baseline characteristics of the study population.

Variables	Total	Total			Men					Women				
		Men	Women	*p*	Q1	Q2	Q3	Q4	Trend *p*	Q1	Q2	Q3	Q4	Trend *p*
Subjects (*n*)	1027	651	376		163	163	163	162		94	94	94	94	
Age (years)^a^	45.3 (7.9)	45.8 (8.0)	44.3 (7.7)	0.005	46.1 (8.1)	47.2 (8.1)	44.3 (7.2)	45.4 (8.3)	0.067	45.3 (8.4)	45.6 (7.6)	44.3 (7.3)	42.2 (7.1)	0.003
BMI (kg/m^2^)^a^	23.2 (2.4)	24.0 (2.0)	21.7 (2.4)	< 0.001	25.2 (1.4)	24.5 (1.8)	23.7 (1.9)	22.6 (2.1)	< 0.001	23.5 (2.2)	22.0 (2.0)	21.7 (1.8)	19.7 (1.7)	< 0.001
Skeletal muscle mass (kg)^a^	27.5 (5.7)	31.2 (3.3)	21.1 (2.4)	< 0.001	29.8 (2.4)	31.0 (3.1)	31.9 (3.4)	32.2 (3.5)	< 0.001	20.1 (2.3)	20.7 (2.3)	21.7 (2.5)	21.7 (2.4)	< 0.001
SMI (%)^a^	41.6 (3.8)	43.6 (2.5)	38.0 (3.0)	< 0.001	40.4 (1.3)	42.8 (0.5)	44.5 (0.5)	46.9 (1.2)	< 0.001	34.3 (1.3)	36.9 (0.5)	38.9 (0.7)	41.9 (1.4)	< 0.001
Fat mass (kg)^a^	16.3 (4.1)	16.1 (4.0)	16.6 (4.3)	0.053	20.4 (2.5)	17.3 (2.3)	15.1 (2.1)	11.6 (2.5)	< 0.001	21.2 (3.4)	17.8 (2.6)	15.7 (2.2)	11.8 (2.4)	< 0.001
Days of vigorous physical activity (per week)^a^	0.9 (1.4)	1.0 (1.4)	0.8 (1.4)	0.021	0.8 (1.4)	0.7 (1.1)	0.9 (1.3)	1.5 (1.7)	< 0.001	0.7 (1.2)	0.6 (1.2)	0.9 (1.4)	0.9 (1.7)	0.131
Smoking				< 0.001					0.114					0.830
Current	130 (12.7)	124 (19.0)	6 (1.6)		24 (14.7)	32 (19.6)	32 (19.6)	36 (22.2)		0 (0.0)	0 (0.0)	1 (1.1)	5 (5.3)	
Former	228 (22.2)	204 (31.3)	24 (6.4)		48 (29.4)	53 (32.5)	50 (30.7)	53 (32.7)		3 (3.2)	4 (4.3)	6 (6.4)	11 (11.7)	
Never	381 (37.1)	120 (18.4)	261 (69.4)		28 (17.2)	28 (17.2)	33 (220.2)	31 (19.1)		67 (71.3)	65 (69.1)	70 (74.5)	59 (62.8)	
Unknown	288 (28.0)	203 (31.2)	85 (22.6)		63 (38.7)	50 (30.7)	48 (29.4)	42 (25.9)		24 (25.5)	25 (26.6)	17 (18.1)	19 (20.2)	
Hypertension (%)	117 (11.4)	94 (14.4)	23 (6.1)	< 0.001	27 (16.6)	30 (18.4)	22 (13.5)	15 (9.3)	0.030	7 (8.5)	1 (1.2)	8 (9.8)	4 (4.9)	0.386
Insulin (uIU/mL)^b^	4.9 (3.5–6.8)	5.3 (3.8–7.6)	4.5 (3.0–5.9)	< 0.001	6.7 (5.0–9.2)	5.9 (4.3–8.1)	5.1 (3.7–6.9)	4.0 (2.8–5.2)	< 0.001	5.4 (4.2–7.0)	4.6 (3.6–5.6)	4.1 (2.6–5.7)	3.4 (2.6–5.0)	< 0.001
Glucose (mg/dL)^b^	93.0 (88.0–99.0)	95.0 (90.0–102.0)	90.0 (86.0–95.0)	< 0.001	96.0 (90.0–103.0)	96.0 (91.0–102.5)	95.0 (89.0–102.0)	94.5 (90.0–99.0)	0.029	92.0 (88.0–98.0)	90.5 (87.0–94.8)	89.0 (85.2–94.0)	89.0 (85.0–93.0)	< 0.001
Triglyceride (mg/dL)^b^	96.0 (70.0–137.5)	111.0 (81.0–156.0)	79.0 (58.0–108.2)	< 0.001	127.0 (94.0–172.5)	125.0 (91.0–170.0)	106.0 (83.0–164.0)	84.0 (67.0–117.0)	< 0.001	96.5 (69.0–126.8)	83.5 (63.2–117.2)	74.5 (53.2–93.0)	68.5 (51.0–84.8)	< 0.001
ALT (U/L)^b^	17.0 (13.0–24.0)	20.0 (15.0–28.0)	13.0 (10.0–16.0)	< 0.001	24.0 (18.0–30.0)	23.0 (16.0–30.0)	19.0 (14.0–27.0)	18.0 (14.0–23.0)	< 0.001	13.0 (10.0–17.0)	12.5 (10.0–17.0)	14.0 (10.0–16.0)	12.0 (9.0–15.0)	0.040
Total calorie (kcal/day)^b^	1375.6 (1035.2–1763.8)	1457.8 (1174.8–1814.8)	1256.7 (885.2–1680.6)	< 0.001	1576.6 (1239.3–1844.9)	1401.4 (888.5–1776.8)	1413.2 (1176.6–1688.2)	1524.7 (1177.3–1986.7)	0.650	1249.7 (980.3–1813.5)	1260.8 (927.3–1602.7)	1256.7 (823.3–1571.9)	1296.0 (777.6–1681.0)	0.421
Protein (g/day)^b^	45.7 (33.1–61.8)	47.9 (35.5–65.0)	41.5 (30.0–56.2)	< 0.001	52.7 (37.1–70.5)	45.4 (32.6–63.5)	47.9 (38.3–59.6)	49.8 (35.7–66.8)	0.975	42.9 (33.4–59.8)	42.1 (31.8–56.5)	37.3 (28.0–52.8)	43.2 (28.1–57.2)	0.293
Fat (g/day)^b^	25.0 (16.0–37.6)	26.9 (16.7–38.1)	23.0 (14.5–36.2)	0.006	31.1 (20.2–41.3)	23.4 (12.8–36.0)	28.4 (18.2–37.6)	24.9 (17.9–38.1)	0.933	24.3 (15.7–36.4)	22.8 (13.0–32.0)	19.6 (13.3–34.5)	24.6 (15.9–37.5)	0.793
Carbohydrate (g/day)^b^	237.8 (168.7–305.4)	249.7 (188.2–313.5)	205.8 (147.3–292.7)	< 0.001	255.6 (223.7–312.2)	239.4 (152.8–298.6)	245.1 (175.3–292.0)	267.6 (205.4–328.2)	0.371	214.0 (159.2–300.7)	202.9 (145.5–271.8)	195.7 (157.9–275.4)	229.6 (125.6–299.1)	0.523
Fibre (g/day)^b^	3.3 (2.2–4.9)	3.4 (2.2–4.8)	3.2 (2.1–5.0)	0.571	3.7 (2.3–4.8)	3.4 (2.3–4.8)	3.2 (2.2–4.9)	3.5 (2.2–4.9)	0.742	3.4 (2.4–5.2)	3.4 (2.6–4.8)	3.0 (1.8–4.6)	2.9 (1.9–5.0)	0.172
Quartiles (Q) of SMI				< 0.001										
Q1	257 (25.0)	20 (3.1)	237 (63.0)											
Q2	257 (25.0)	152 (23.3)	105 (27.9)											
Q3	257 (25.0)	231 (35.5)	26 (6.9)											
Q4	256 (25.0)	248 (38.1)	8 (2.1)											

*Note:* Continuous variables were compared using *t*‐test or Mann–Whitney *U* test. The frequencies of categorical variables were compared using chi‐squared test. For trend test, linear regression (parametric) or Jonkheere–Terpstara (non‐parametric) test was used for continuous variables and Cochran–Armitage trend test for categorical variables.

Abbreviations: ALT, alanine aminotransferase; BMI, body mass index; SMI, skeletal muscle index.

^a^ Data are expressed as mean (standard deviation).

^b^ Data are expressed as median (interquartile range).

### 3.2 Sex‐Specific Differences in Microbial Taxa Associated With SMI

When compared between the SMI groups, the alpha diversity (i.e., within‐sample diversity) showed no significant differences in the Shannon and Simpson indices in both sexes (Figure S1). However, the observed species richness was markedly different between the Q1 and Q4 groups in men (*p* = 0.026). In the case of beta diversity (i.e., diversity across samples), only the Jaccard index showed differences among men (*p* = 0.020) (Figure S2).

**FIGURE 2:**
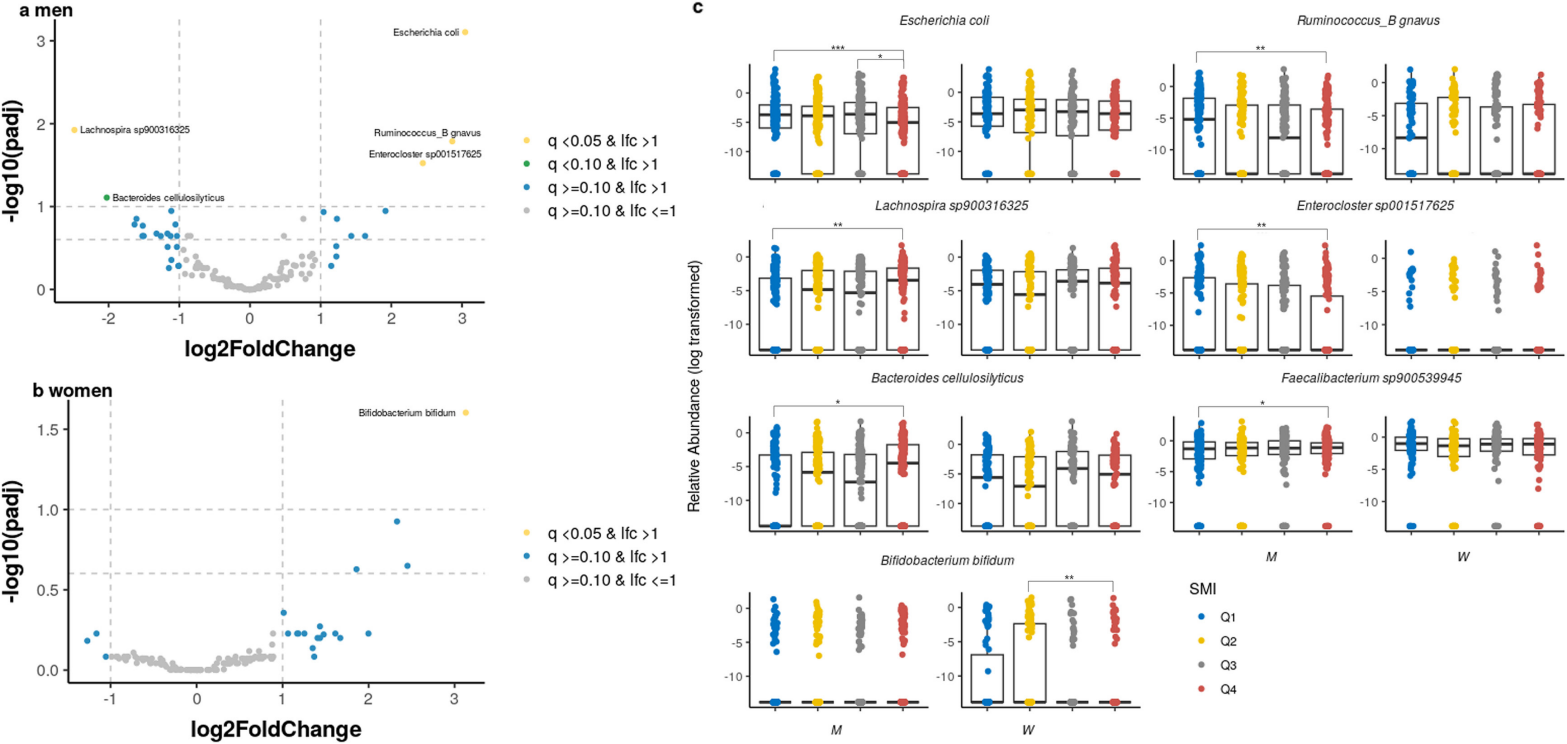
Differentially abundant taxa based on skeletal muscle mass index. Volcano plots of the estimated log2 fold difference in species abundance between the reference group (Q4) and (a) Q1 for men and (b) Q2 for women. Species that were differentially abundant at *q* < 0.10 in men and women were selected, and (c) relative abundances across SMI quartiles were displayed by sex (***p < 0.01, **p < 0.05, and *p < 0.10). Results were obtained from differential abundance analysis using a linear differential abundance analysis (LinDA) adjusted for age, body mass index (BMI), and days of vigorous physical activity for men and age and BMI for women. Taxa with positive log2 fold‐change values were significantly more abundant (enriched) in the comparison group, whereas those with negative log2 fold‐change values were more abundant in the reference (Q4) group. The Benjamini–Hochberg adjusted *p*‐value is denoted as *q*, and the names of species that are differentially abundant at *q* < 0.10 are given. See Table S1 for a full list of differentially abundant species with a *q*‐value of < 0.25.

After adjusting for covariates, LinDA was used to identify the microbial taxa that were significantly different from the other groups based on the highest SMI group (Q4). A total of 2430 species were identified, and after applying filtering options, 1792 features were filtered in men and 1774 in women. Consequently, 638 and 656 features were included for further analyses in men and women, respectively. Compared with the Q4 group, in men, six species were significantly associated with the Q1 group (*q* < 0.1), with four of these species showing *q*‐values < 0.05. In the Q1 group, higher levels were associated with *Escherichia coli* , *Ruminococcus_B gnavus* and *Enterocloster sp001517625*, whereas lower levels were associated with *Lachnospira sp900316325*, *Bacteroides cellulosilyticus* and *Faecalibacterium sp900539945* compared to the Q4 group (Figure 2a and Table S1). These species were not significantly represented in the analyses of women. Instead, the abundances of *Bifidobacterium bifidum* were significantly higher in the Q2 group than in the Q4 group (*q* < 0.05) (Figure 2b and Table S1). For species with a significant difference in the analysis of men and women, box plots were created to compare the distribution of groups by sex (Figure 2c). Notably, *Escherichia coli* and *Ruminococcus_B gnavus* showed a similar pattern in both sexes, with lower abundance in the Q4 group compared to the Q1 or Q2 groups. Likewise, *Lachnospira sp900316325* and *Bacteroides cellulosilyticus* were more abundant in Q4 than Q2 group in women.

We performed subset analyses within quartiles of similar age to identify age‐independent associations (Table S2). In men, *Escherichia coli* was significantly associated with both the age under 45 and 45–64 age groups. However, in women, no species showed significant differences in the age under 45, while additional species, including *Bifidobacterium bifidum* , were identified only in the 45–64 age group.

### 3.3 Sex‐Specific Differences in Functional Potential of Microbiota Associated With SMI

Differential abundance analysis of functional pathways from metagenomic data was also performed by comparing the highest abundance group with other groups. From the HUMAnN3 results, 536 pathways were identified. After filtering out those present in less than 10% of subjects and with a relative abundance below 0.1%, 170 pathways were included for analysis in men and 177 pathways in women. When comparing the Q4 and Q1 groups in the differential abundance analysis, 11 metabolic pathways were found to be significantly more abundant in the Q1 group in men (*q* < 0.1) (Table 2). Notably, most of the significantly different metabolic pathways were involved in the amino acid biosynthesis (MET‐SAM‐PWY, *q* = 0.060; METSYN‐PWY, *q* = 0.060, SER‐GLYSYN‐PWY, *q* = 0.060; PWY‐5347, *q* = 0.063; P4‐PWY, *q* = 0.090). Differences in abundance were also observed in the degradation or salvage of purine nucleotides (PWY‐6353, *q* = 0.060; PWY66‐409, *q* = 0.060), guanosine nucleotides (PWY‐6608, *q* = 0.060) and adenosine nucleotides (SALVADEHYPOX‐PWY, *q* = 0.073). Additionally, the pathways related to carboxylic acid degradation (*N*‐acetylneuraminate) and involved in isoprene biosynthesis were significantly different when comparing Q1 and Q4 groups. Comparison results with the Q3 group also showed that pathways related to amino acid biosynthesis were significant (Table S3). On the other hand, in women, there was no significant pathways in the comparison of the Q1 and Q4 groups, and all significant pathways (*q* < 0.1) appeared in the comparison of Q2 and Q4 groups. In particular, the pathways with *q* < 0.05 were found to be directly or indirectly related to energy production, such as lactic acid fermentation (ANAEROFRUCAT‐PWY, *q* = 0.014), pentose phosphate pathway (PENTOSE‐P‐PWY, *q* = 0.014) and glycolysis (PWY‐5484, *q* = 0.014; GLYCOLYSIS, *q* = 0.014; PWY‐6901, *q* = 0.014).

**TABLE 2:** Associations of skeletal muscle mass index quartiles and bacterial functional pathways in men.

Pathway	log2FoldChange	lfcSE	*p*	*q*
MET‐SAM‐PWY: superpathway of *S*‐adenosyl‐L‐methionine biosynthesis	0.42	0.14	0.003	0.060
METSYN‐PWY: superpathway of L‐homoserine and L‐methionine biosynthesis	0.44	0.15	0.003	0.060
P441‐PWY: superpathway of *N*‐acetylneuraminate degradation	0.43	0.14	0.002	0.060
PWY‐6270: isoprene biosynthesis I	0.20	0.06	0.002	0.060
PWY‐6353: purine nucleotides degradation II (aerobic)	0.42	0.12	0.001	0.060
PWY‐6608: guanosine nucleotides degradation III	0.38	0.12	0.002	0.060
PWY66‐409: superpathway of purine nucleotide salvage	0.52	0.17	0.002	0.060
SER‐GLYSYN‐PWY: superpathway of L‐serine and glycine biosynthesis I	0.20	0.06	0.001	0.060
PWY‐5347: superpathway of L‐methionine biosynthesis (transsulfuration)	0.41	0.14	0.003	0.063
SALVADEHYPOX‐PWY: adenosine nucleotides degradation II	0.43	0.15	0.004	0.073
P4‐PWY: superpathway of L‐lysine, L‐threonine and L‐methionine biosynthesis I	0.53	0.19	0.006	0.090

*Note:* The models were adjusted for age, body mass index, and days of vigorous physical activity. The highest quartile (Q4) group was set as the reference and compared with the lowest quartile (Q1) group. Only *q*‐values of < 0.10 are shown.

Abbreviations: lfcSE, standard error estimate for the log2 fold change estimate; log2FoldChange, log2 fold change estimate; *p*, *p*‐value; *q*, adjusted *p*‐value.

### 3.4 Identification of Key Microbial Species Involved in Significant SMI‐Associated Metabolic Pathways

**FIGURE 3:**
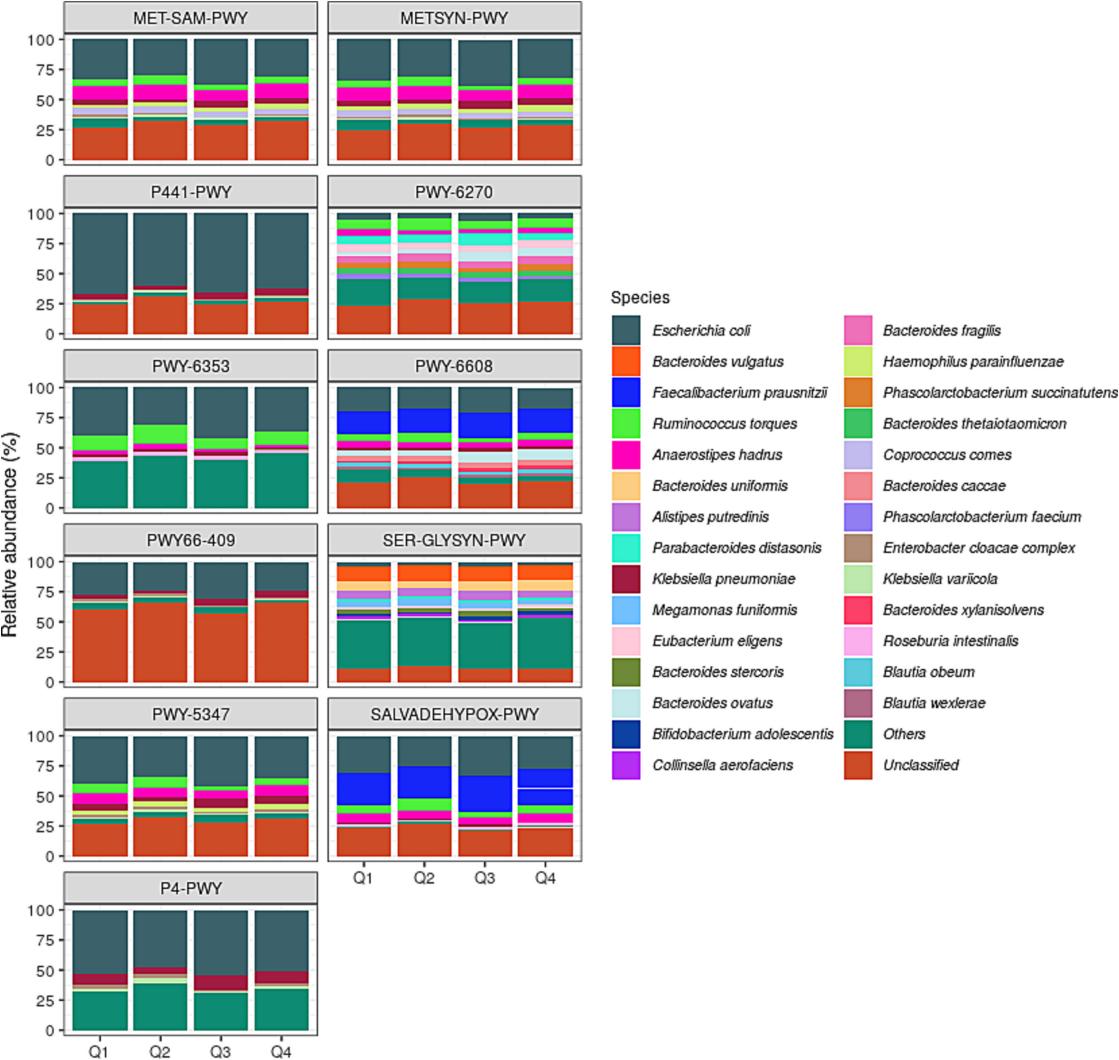
Contributive diversity of microbial taxa to functional potentials of gut microbiome. The bar graphs represent the major contributing species to pathways in the linear differential abundance analysis (LinDA), which were significantly different (adjusted *p*‐value < 0.1) for the Q1 group compared with the Q4 group. Each set of stacked bars represents the total abundance (in percent) of species in the group. Abbreviations: MET‐SAM‐PWY, superpathway of *S*‐adenosyl‐L‐methionine biosynthesis; METSYN‐PWY, superpathway of L‐homoserine and L‐methionine biosynthesis; P441‐PWY, superpathway of *N*‐acetylneuraminate degradation; P4‐PWY, superpathway of L‐lysine, L‐threonine and L‐methionine biosynthesis I; PWY0‐781, aspartate superpathway; PWY‐5347, superpathway of L‐methionine biosynthesis (transsulfuration); PWY‐6270, isoprene biosynthesis I; PWY‐6353, purine nucleotides degradation II (aerobic); PWY‐6608, guanosine nucleotides degradation III; SALVADEHYPOX‐PWY, adenosine nucleotides degradation II.

We investigated the microbial species that primarily contributed to the 11 SMI‐associated functional pathways identified in Table 2. Figure 3 shows the top 30 contributing species for each pathway per SMI group, with *Escherichia coli* being the largest contributor to most pathways. The second largest contributors after *Escherichia coli* included *Bacteroides vulgatus* for superpathway of L‐serine and glycine biosynthesis I (SER‐GLYSYN‐PWY); *Faecalibacterium prausnitzii* for guanosine nucleotides degradation III (PWY‐6608) and adenosine nucleotides (SALVADEHYPOX‐PWY); *Ruminococcus torques* for purine nucleotides degradation (PWY‐6353) and isoprene biosynthesis I (PWY‐6270); *Anaerostipes hadrus* for superpathway of S‐adenosyl‐L‐methionine biosynthesis (MET‐SAM‐PWY), superpathway of L‐homoserine and L‐methionine biosynthesis (METSYN‐PWY) and superpathway of L‐methionine biosynthesis (PWY‐5347); and *Klebsiella pneumoniae* for superpathway of L‐lysine, L‐threonine and L‐methionine biosynthesis I (P4‐PWY).

**FIGURE 4:**
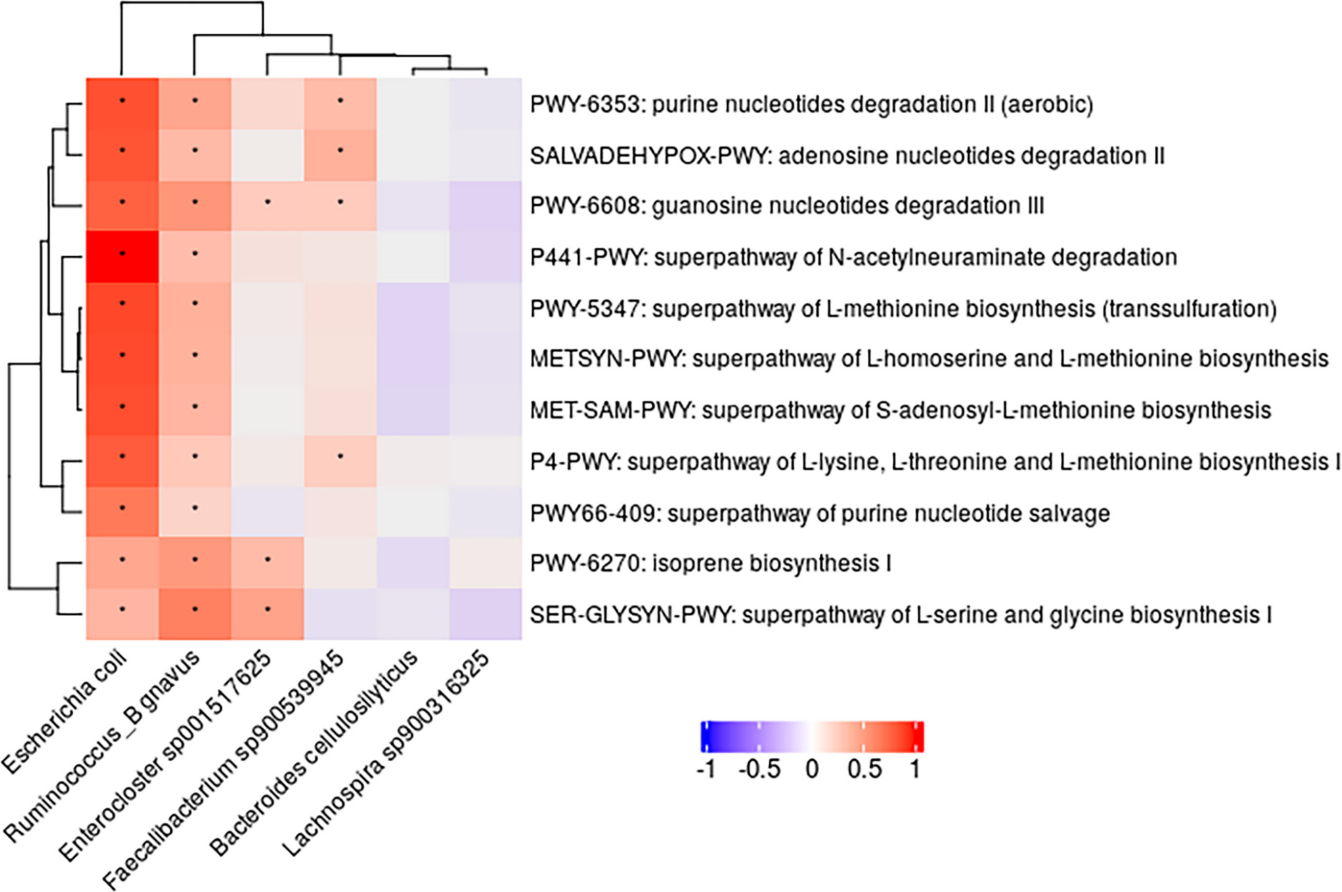
Correlation heatmap of differentially abundant species and skeletal muscle index‐associated bacterial functional potentials among men. Heatmap of Spearman's correlation analysis between the gut microbiota and microbial pathways in men. Pathways in the linear differential abundance analysis (LinDA) that were significantly different compared to Q4 groups, with an adjusted *p*‐value of < 0.1, were selected (see Table 2). Similarly, species with adjusted *p*‐values < 0.1 were selected (see Table S1). *Statistically significant Spearman correlations (adjusted *p*‐value < 0.05) after correction for multiple testing using Benjamini–Hochberg adjustment.

In addition, we plotted a heatmap of the correlations to determine how the six SMI‐associated species identified in the LinDA results were related to the 11 pathways (Figure 4). Among them, *Escherichia coli* was positively correlated with all 11 pathways (adjusted *p* < 0.05). Following *Escherichia coli* , *Ruminococcus_B gnavus, Enterocluster sp001517625* and *Faecalibacterium sp900539945* were positively correlated with certain pathways.

### 3.5 Differential Analysis for Metabolites Across SMI Groups

**FIGURE 5:**
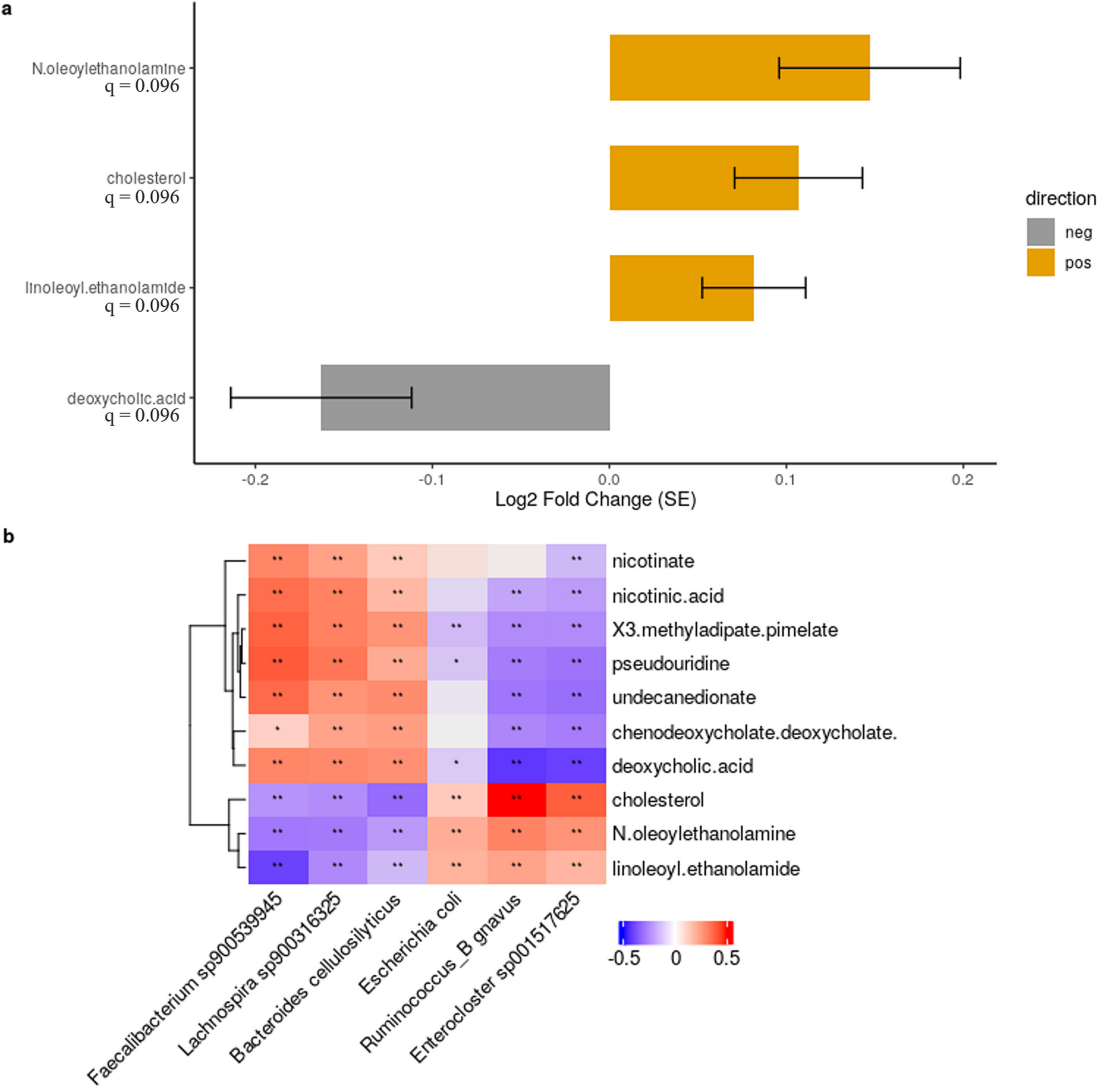
Differentially abundant metabolites based on skeletal muscle mass index in men. Plots showing the (a) estimated log2 fold difference in the abundance of metabolites and (b) Spearman's correlation between the species and top 10 differentially abundant metabolites in men. Results of metabolites were obtained from differential abundance analysis using a linear differential abundance analysis (LinDA) compared to the Q4 group, adjusted for age, body mass index (BMI) and days of vigorous physical activity. Metabolites with positive log2 fold‐change values (yellow bars) were significantly more abundant (enriched) in the Q1 than the Q4 group, and those with negative log2 fold‐change values (grey bars) were enriched in the Q4 group. Error bars represent standard error, and the Benjamini–Hochberg adjusted *p*‐value is denoted as *q*. The log2 fold change estimates are as follows: *N*‐oleoylethanolamine: 0.15, cholesterol: 0.11, linoleoyl ethanolamide: 0.08, deoxycholic acid: ‐0.16. For Spearman correlation analysis, species that were significantly different in the Q1 group compared to the Q4 group were selected (adjusted *p*‐value < 0.1). Significant correlation between species and metabolites (*p < 0.05, and **p < 0.01).

Using the 80 predicted metabolite profiles from the MelonnPan results, we analysed the differential metabolites based on the Q4 groups. After filtering to remove relative abundances below 0.01% in more than 5% of the samples, 72 samples remained for analysis in men and women. Metabolites that significantly differed between the groups were identified only in men, with one in the Q2 group and four in Q1 with *q* < 0.1. The Q1 group had higher levels of cholesterol, linoleoyl ethanolamide (LEA), and *N*‐oleoylethanolamine (OEA) and lower levels of deoxycholic acid (DCA). OEA was also found to be abundant in Q2 (Figure 5 and Table S4). In a heatmap of the correlation between six SMI‐related species and the top 10 metabolites found in differential abundance analysis, cholesterol, LEA, and OEA were positively correlated with *Escherichia coli* , *Ruminococcus _B gnavus*, and *Enterocluster sp001517625*.

## 4 Discussion

This study investigated the association between SMI and microbial taxa and functional potential by focusing on sex‐specific disparities among Korean adults. Our findings highlight remarkable differences in microbial functions associated with SMI in men and women. Notably, in men, the metabolic pathways involved in purine degradation, salvage and amino acid biosynthesis were more abundant in the group with the lowest SMI, while in women, metabolic pathways related to energy production were more abundant in the group with the second lowest SMI. Moreover, differences in the abundance of species were extensively observed between the lowest and highest SMI groups in men, with some of these species showing a positive correlation with functional potentials.

The functional potential of gut microbes identified in men may reflect both skeletal muscle wasting and compensatory responses. We identified enhanced microbial pathways related to amino acid synthesis and N‐acetylneuraminate (Neu5Ac) degradation. The higher abundance of amino acid biosynthesis is crucial for maintaining and building skeletal muscle mass [28]. Additionally, elevated Neu5Ac degradation, which produces key components necessary for muscle regeneration, supports this compensatory role [28]. These findings suggest that the gut microbiota may contribute to muscle synthesis and regeneration, potentially as a response to muscle loss, explaining the prominence of these pathways in individuals with lower SMI. Furthermore, purine metabolism, essential for cellular functions such as growth, energy metabolism and neurotransmission [29], is also linked to muscle health. Purine degradation, which occurs during muscle atrophy, especially under conditions like intense contraction or hypoxia, suggests a connection to microbial activity [30]. The presence of microbial pathways associated with purine degradation in individuals with lower skeletal muscle mass indicates that gut microbes may contribute to or respond to muscle catabolism, reflecting the stress and metabolic demands placed on muscle tissue.

In women, microbial metabolic pathways also appear to reflect those pathways associated with muscle production. Carbohydrate metabolism including glycolytic activity is important for generating ATP during high‐intensity exercise, sustaining muscle contractions and preventing muscle breakdown [31]. A previous study suggests that skeletal muscle hypertrophy involves a metabolic shift, where glucose carbon is diverted to anabolic pathways to support muscle mass building [32]. This was further supported by a review that found 10 metabolites associated with muscle hypertrophy were directly linked to glycolysis and interconnected anabolic pathways, such as the pentose phosphate pathway. Just as the gut microbiota may need to actively produce amino acids to support skeletal muscle synthesis in muscle‐deficient conditions, increased carbohydrate metabolism might also play a role in the maintenance and production of muscle in women. In addition, some microbial functions are shared between women and men, suggesting common functions of the microbiota that may contribute to skeletal muscle maintenance.

Results from the differential abundance analysis of microbial taxa varied between men and women. Men with the lowest SMI had a higher abundance of *Escherichia coli*, *Ruminococcus_B gnavus*, and *Enterocloster sp001517625*. However, this trend was not observed in women. Specifically, *Escherichia coli* , which has been implicated in the regulation of glucose homeostasis and energy metabolism and may participate in the pathogenesis of obesity and type 2 diabetes [33], was positively correlated with significantly enriched functional pathways and is a major contributor to most of these functions. On the other hand, *Bifidobacterium, Lachnospira, Bacteroides*, and *Faecalibacterium*, identified in our results, are involved in the production of short‐chain fatty acids (SCFAs) through the fermentation of dietary fibers and are known to be protective bacteria in several diseases [34]. However, further research is needed to elucidate the specific mechanisms by which these gut microbes influence muscle health, as this area remains poorly understood.

Our findings revealed differences in microbial function and species associated with SMI in men and women. These differences might be explained by variations in gut microbiota composition influenced by sex hormones, which, along with the hormones themselves, may contribute to the observed differences in muscle mass [12, 13]. Although lifestyle factors such as smoking and physical activity were considered in this study, other environmental factors that may vary by sex could also influence the composition and function of the gut microbiota. Future studies should explore these variables to further refine our understanding of the microbiome's role in muscle health across sexes. Integrating these factors could lead to more precise interventions and personalized approaches to promoting muscle health through microbiome optimization.

Additionally, our study explored the association between predicted metabolomic profiling of the gut microbiome and SMI, particularly highlighting the microbiome‐muscle axis via bile acids. Cholesterol, synthesized in the liver as a precursor to primary bile acid and converted to secondary bile acids such as DCA by gram‐positive bacteria in the small intestine, acts as a signaling molecule that regulates insulin sensitivity, energy expenditure, lipid accumulation and glucose homeostasis through various receptors [35]. Interestingly, the gut microbiota of patients with cirrhosis, a known risk factor for sarcopenia, showed a dysbiosis with a lower prevalence of cholesterol‐metabolizing bacteria (*Lachnospiraceae* and *Ruminococcaceae*) and a higher abundance of potentially pathogenic bacteria (*Enterobacteriaceae*) compared to controls [36]. Other studies have shown that the dysfunctional microbial metabolism of bile acids leads to decreased skeletal muscle mass, muscle fiber size, strength, and lower DCA levels [37]. In addition, given the roles of OEA and LEA in lipid metabolism and anti‐inflammatory responses and appetite regulation, we found that altered levels of these metabolites could potentially be associated with sarcopenia [38]. However, more research is needed to clarify this relationship.

Our results showed, consistentwith previous findings in the 16S rRNA gene analysis, that there are sex‐specific differences in the abundance and function of gut microbiota [14]. A key aspect of this study is the validation of our earlier findings using shotgun sequencing, which provides higher precision at the species level compared to 16S rRNA analysis. This method allowed us to identify additional skeletal muscle‐associated species, such as *Escherichia coli* , that were not previously reported. Furthermore, functional profiles obtained from metagenomic shotgun sequencing supported predicted pathways from earlier studies, improved our understanding of the functional relevance to skeletal muscle. While metagenomic sequencing offers significant advantages, including more detailed and comprehensive data, it is also considerably more expensive and time‐consuming. These limitations can affect the feasibility of metagenomic approaches, particularly in large‐scale studies or resource‐limited settings. Therefore, these constraints should be carefully considered when designing studies and interpreting results, as the choice of sequencing method can significantly influence the scope and depth of the data obtained.

In this study, we assessed muscle mass using BIA, a convenient method shown to correlate with dual‐energy X‐ray absorptiometry in Koreans [39]. While BIA can overestimate muscle mass compared to gold‐standard methods like CT or MRI, equations have been refined to improve accuracy. Most validation studies, however, have focused on European populations and older adults. A study by Lee et al. in a Korean cohort aged 20–90 found that differences between BIA and DXA were influenced by BMI, sex, and body fat, but not age [40]. This suggests that, although age is often included in BIA equations, it may not be a significant factor in muscle mass estimations for Koreans. As a result, age‐related estimation errors in BIA muscle mass were not considered in this study. Additionally, diagnosing sarcopenia requires evaluating not just muscle mass but also muscle strength and physical performance, such as grip strength or walking speed, which were not measured here. Therefore, further studies incorporating more accurate muscle mass measurements and assessments of muscle strength are needed.

This study has some limitations. First, the cross‐sectional design requires precludes causal inferences. Therefore, longitudinal studies, animal model experiments and randomized controlled trials are needed to determine potential causal relationships. Second, because we did not conduct a meta‐transcriptome analysis or measure metabolites, the metabolites inferred from our results should be considered with caution. Nonetheless, our findings can be explained based on skeletal muscle physiology, with some of our results validated in mouse experiments. Third, because we excluded several participants, the generalizability of our findings to sarcopenia patients may be limited. By excluding participants with severe comorbidities, our study focuses on the relationship between gut microbiota and skeletal muscle mass in a relatively healthy population. This approach allows us to isolate the effects of gut microbiota on muscle mass without the confounding influence of these conditions. While this exclusion criterion may limit the generalizability of our findings to individuals with such comorbidities, it strengthens the internal validity of our study by reducing potential confounding factors. Additionally, we focused on adults under 65, excluding those over 65 due to small sample sizes. While most sarcopenia studies concentrate on older adults [1], our study highlights the gut microbiome as a potential biomarker for muscle loss in younger adults. Since muscle mass loss often begins after age 25, early identification of at‐risk individuals may be crucial for prevention [41]. Fourth, using questionnaires to assess physical activity and dietary intake may introduce biases like underreporting and recall errors. However, these tools are widely used in epidemiological studies for their cost‐effectiveness and feasibility, especially in large populations. Importantly, these variables were used as covariates rather than primary outcomes, so the required precision is lower. Despite their limitations, we believe that the questionnaires provide sufficiently reliable data for adjusting for potential confounders in the analysis.

In conclusion, this study elucidated sex‐specific differences in the microbial taxa and functional potential associated with SMI. Insights into specific microbial species and their functions related to SMI provide opportunities for personalized strategies to enhance muscle function, prevent age‐related muscle loss, and improve overall physical health and quality of life.

## Ethics Statement

The study protocol was conducted in accordance with the Declaration of Helsinki and approved by the Institutional Review Board (IRB) of Seoul National University (SNU 24‐04‐127). Written informed consent was obtained from all participants after explaining all possible consequences of the study.

## Conflicts of Interest

The authors declare no conflicts of interest.

## Supporting information


**Figure S1** Alpha diversity of the gut microbiota among the skeletal muscle mass index groups. Alpha diversity metrics, observed number, and Shannon and Simpson diversity indices are shown as boxplots across quartiles of skeletal muscle mass index for (a, c, e) men and (b, d, f) women. Statistics were calculated using linear regressions with these measures as response variables and adjusted for age, body mass index (BMI), and days of vigorous physical activity for men and age and BMI for women. Q4 was used as a reference. *p < 0.05. Boxes represent the interquartile range, and the horizontal line inside the box defines the median values.
**Figure S2.** Principal coordinate analyses (PCoA) based on beta diversity among quartiles of skeletal muscle mass index groups. Principal coordinate analysis was performed on the matrices of (a) Bray–Curtis dissimilarity in men, (b) Jaccard distance in men, (c) Bray–Curtis dissimilarity in women, and (d) Jaccard distance in women. Statistical significance between the skeletal muscle index groups was calculated using pairwise PERMANOVA with 999 permutations adjusted for age, body mass index (BMI), and days of vigorous physical activity for men and age and BMI for women. The percentage of variability explained by the first two PCoA (Axis‐1 and Axis‐2) is represented by the x‐ and y‐axes, respectively, and the ellipses represent the 95% confidence intervals. Each data point represents a microbial community sample colored according to the skeletal muscle mass index groups.
**Table S1.** Association of skeletal muscle index groups and taxa.
**Table S2.** Association of skeletal muscle index groups and taxa, stratified by age group.
**Table S3.** Associations of skeletal muscle mass index quartiles and bacterial functional pathways.
**Table S4.** Associations of skeletal muscle mass index groups and bacterial metabolite in men.
