# Supplementary material for: Metagenomic Analysis Identifies Sex‐Related Gut Microbial Functions and Bacterial Taxa Associated With Skeletal Muscle Mass
Source: J Cachexia Sarcopenia Muscle. 2024 Nov 19;16(1):e13636. doi: 10.1002/jcsm.13636 (PMC11670178; doi:10.1002/jcsm.13636)
Supplement: Supplementary file 1 — Figure S1 Alpha diversity of the gut microbiota among the skeletal muscle mass index groups. Alpha diversity metrics, observed number, and Shannon and Simpson diversity indices are shown as boxplots across quartiles of skeletal muscle mass index for (a, c, e) men and (b, d, f) women. Statistics were calculated using linear regressions with these measures as response variables and adjusted for age, body mass index (BMI), and days of vigorous physical activity for men and age and BMI for women. Q4 was used as a reference. *p < 0.05. Boxes represent the interquartile range, and the horizontal line inside the box defines the median values. Figure S2. Principal coordinate analyses (PCoA) based on beta diversity among quartiles of skeletal muscle mass index groups. Principal coordinate analysis was performed on the matrices of (a) Bray–Curtis dissimilarity in men, (b) Jaccard distance in men, (c) Bray–Curtis dissimilarity in women, and (d) Jaccard distance in women. Statistical significance between the skeletal muscle index groups was calculated using pairwise PERMANOVA with 999 permutations adjusted for age, body mass index (BMI), and days of vigorous physical activity for men and age and BMI for women. The percentage of variability explained by the first two PCoA (Axis‐1 and Axis‐2) is represented by the x‐ and y‐axes, respectively, and the ellipses represent the 95% confidence intervals. Each data point represents a microbial community sample colored according to the skeletal muscle mass index groups. Table S1. Association of skeletal muscle index groups and taxa. Table S2. Association of skeletal muscle index groups and taxa, stratified by age group. Table S3. Associations of skeletal muscle mass index quartiles and bacterial functional pathways. Table S4. Associations of skeletal muscle mass index groups and bacterial metabolite in men. [file JCSM-16-e13636-s001.docx]

**Supporting Information**

**Metagenomic Analysis Identifies Sex-Related Gut Microbial Functions and Bacterial Taxa Associated with Skeletal Muscle Mass**

Hang A Park, Joohon Sung, Yoosoo Chang, Seungho Ryu, Kyung Jae Yoon, Hyung-Lae Kim, Han-Na Kim

**Corresponding author**

Han-Na Kim

Department of Clinical Research Design and Evaluation, Samsung Advanced Institute for Health Sciences and Technology, Sungkyunkwan University, 115 Irwon-ro, Gangnam-gu, Seoul 06355, Republic of Korea; Biomedical Statistics Center, Research Institute for Future Medicine, Samsung Medical Center, Gangnam-gu, Seoul 06351, Republic of Korea

Tel: +82-2-3410-2427

Fax: + 82-2-3410-6639

Email: [hanna147942@gmail.com](mailto:hanna147942@gmail.com)


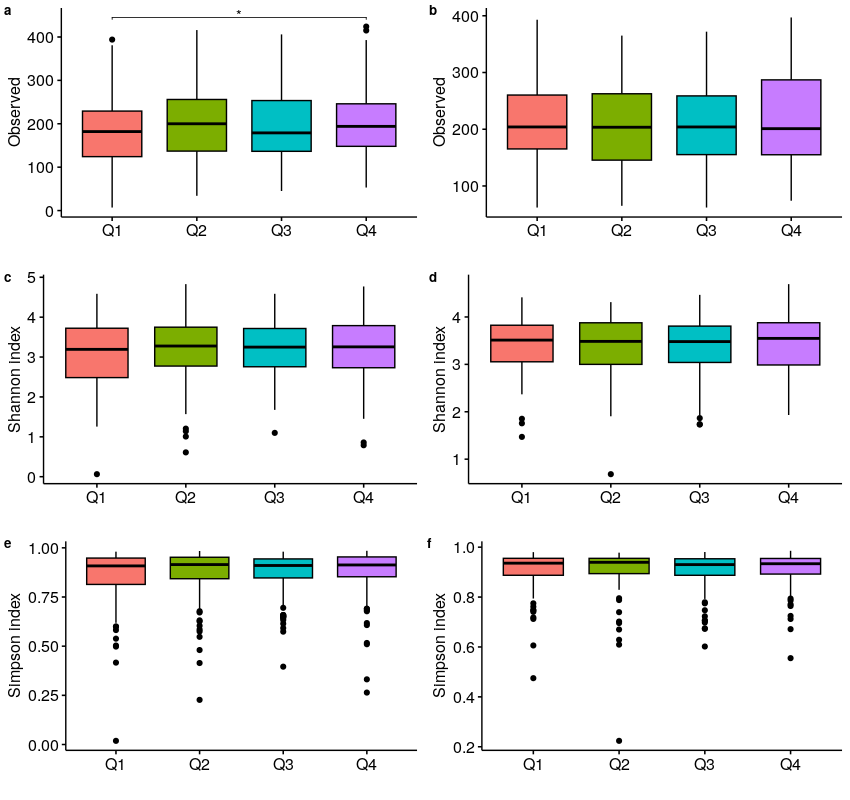


**Fig. S1 Alpha diversity of the gut microbiota among the skeletal muscle mass index groups.** Alpha diversity metrics, observed number, and Shannon and Simpson diversity indices are shown as boxplots across quartiles of skeletal muscle mass index for (a, c, e) men and (b, d, f) women. Statistics were calculated using linear regressions with these measures as response variables and adjusted for age, body mass index (BMI), and days of vigorous physical activity for men and age and BMI for women. Q4 was used as a reference. **p* < 0.05. Boxes represent the interquartile range, and the horizontal line inside the box defines the median values


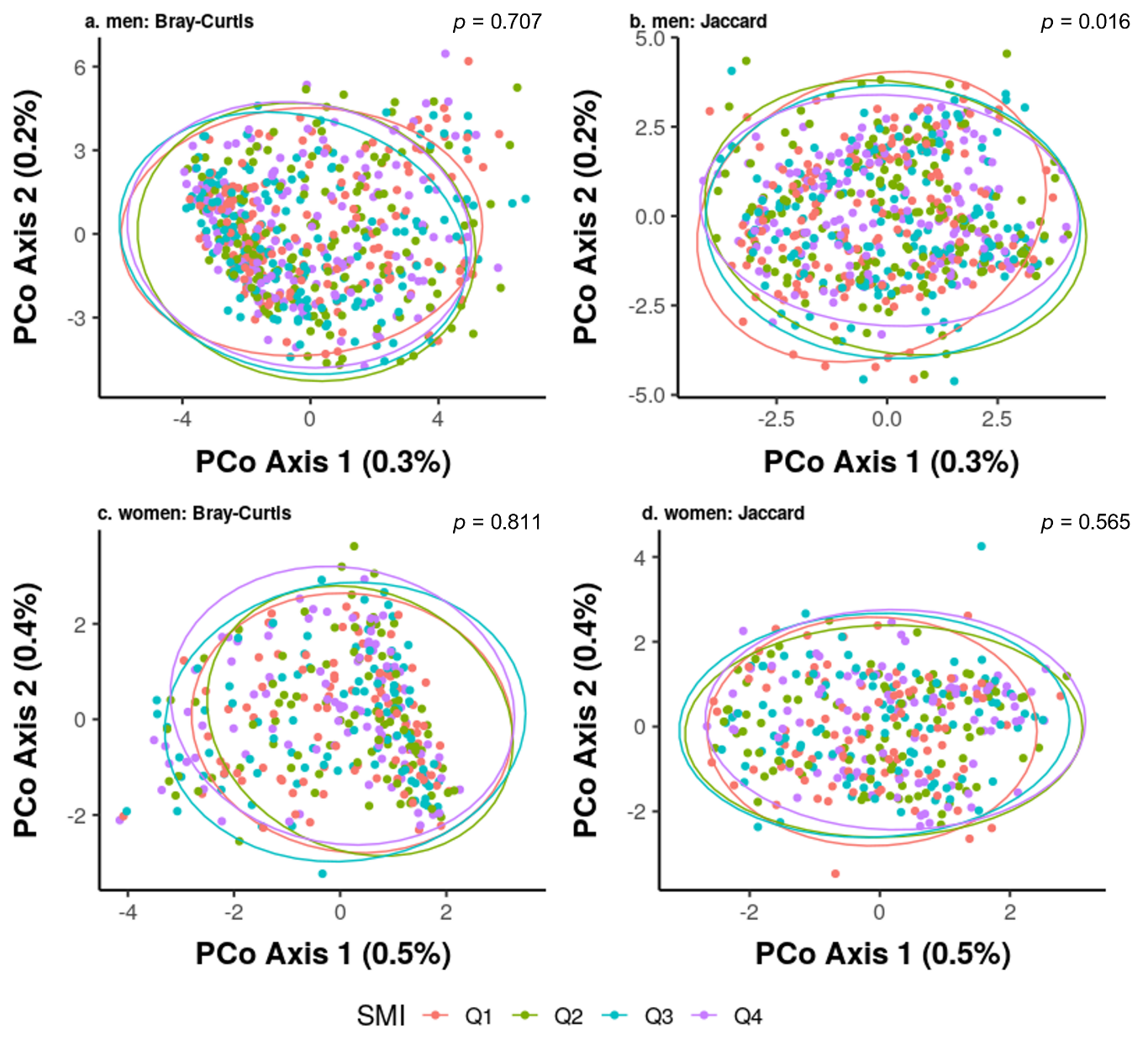


**Fig. S2 Principal coordinate analyses (PCoA) based on beta diversity among quartiles of skeletal muscle mass index groups.** Principal coordinate analysis was performed on the matrices of a) Bray-Curtis dissimilarity in men, b) Jaccard distance in men, c) Bray-Curtis distance in women, and d) Jaccard distance in women. Statistical significance between the skeletal muscle index groups was calculated using pairwise PERMANOVA with 999 permutations adjusted for age, body mass index (BMI), and days of vigorous physical activity for men and age and BMI for women. The percentage of variability explained by the first two PCoA (Axis-1 and Axis-2) is represented by the x- and y-axes, respectively, and the ellipses represent the 95% confidence intervals. Each data point represents a microbial community sample colored according to the skeletal muscle mass index groups.

| **Table S1. Association of skeletal muscle index groups and taxa** | | | | | |
| --- | --- | --- | --- | --- | --- |
| Taxonomy | log2FoldChange | lfcSE | p | q | value |
| **Men** |  |  |  |  |  |
| p_Proteobacteria; c_Gammaproteobacteria; o_Enterobacterales; f_Enterobacteriaceae; g_Escherichia; s_coli | 3.08 | 0.66 | 0.000 | 0.001 | Q1 |
| p_Firmicutes_A; c_Clostridia; o_Lachnospirales; f_Lachnospiraceae; g_Ruminococcus_B; s_gnavus | 2.89 | 0.79 | 0.000 | 0.014 | Q1 |
| p_Firmicutes_A; c_Clostridia; o_Lachnospirales; f_Lachnospiraceae; g_Lachnospira; s_sp900316325 | -2.44 | 0.66 | 0.000 | 0.014 | Q1 |
| p_Proteobacteria; c_Gammaproteobacteria; o_Enterobacterales; f_Enterobacteriaceae; g_Escherichia; s_coli | 2.35 | 0.60 | 0.000 | 0.014 | Q3 |
| p_Firmicutes_A; c_Clostridia; o_Lachnospirales; f_Lachnospiraceae; g_Enterocloster; s_sp001517625 | 2.47 | 0.73 | 0.001 | 0.026 | Q1 |
| p_Bacteroidota; c_Bacteroidia; o_Bacteroidales; f_Bacteroidaceae; g_Bacteroides; s_cellulosilyticus | -2.08 | 0.67 | 0.002 | 0.059 | Q1 |
| p_Firmicutes_A; c_Clostridia; o_Oscillospirales; f_Ruminococcaceae; g_Faecalibacterium; s_sp900539945 | -1.15 | 0.40 | 0.004 | 0.086 | Q1 |
| p_Firmicutes_A; c_Clostridia; o_Lachnospirales; f_Lachnospiraceae; g_Eubacterium_F; s_Eubacterium_F sp003491505 | -1.70 | 0.61 | 0.006 | 0.117 | Q1 |
| p_Firmicutes_C; c_Negativicutes; o_Acidaminococcales; f_Acidaminococcaceae; g_Phascolarctobacterium; s_faecium | 1.86 | 0.69 | 0.007 | 0.127 | Q1 |
| p_Firmicutes_A; c_Clostridia; o_Lachnospirales; f_Lachnospiraceae; g_Bariatricus; s_comes | 1.01 | 0.38 | 0.008 | 0.131 | Q1 |
| p_Firmicutes_A; c_Clostridia; o_Lachnospirales; f_Lachnospiraceae; g_Ruminococcus_D; s_bicirculans | -1.71 | 0.67 | 0.011 | 0.155 | Q1 |
| p_Firmicutes_A; c_Clostridia; o_Lachnospirales; f_Lachnospiraceae; g_Blautia_A; s_wexlerae | 0.74 | 0.29 | 0.012 | 0.160 | Q1 |
| p_Firmicutes_A; c_Clostridia; o_Lachnospirales; f_Lachnospiraceae; g_Clostridium_A; s_leptum | 1.17 | 0.48 | 0.016 | 0.185 | Q1 |
| p_Firmicutes_A; c_Clostridia_A; o_Christensenellales; f_CAG-74; g_UBA11524; s_sp000437595 | -1.49 | 0.63 | 0.018 | 0.189 | Q1 |
| p_Firmicutes_A; c_Clostridia; o_Oscillospirales; f_Ruminococcaceae; g_Faecalibacterium; s_prausnitzii_I | -1.02 | 0.43 | 0.019 | 0.189 | Q1 |
| p_Firmicutes_A; c_Clostridia; o_Oscillospirales; f_Ruminococcaceae; g_Gemmiger; s_qucibialis | -1.18 | 0.51 | 0.021 | 0.199 | Q1 |
| p_Proteobacteria; c_Gammaproteobacteria; o_Enterobacterales; f_Enterobacteriaceae; g_Escherichia; s_coli | 2.02 | 0.64 | 0.002 | 0.208 | Q2 |
| p_Firmicutes_A; c_Clostridia; o_Oscillospirales; f_Ruminococcaceae; g_Faecalibacterium; s_sp900539945 | -1.13 | 0.38 | 0.003 | 0.208 | Q2 |
| p_Bacteroidota; c_Bacteroidia; o_Bacteroidales; f_Rikenellaceae; g_Alistipes; s_finegoldii | -1.30 | 0.58 | 0.026 | 0.232 | Q1 |
| p_Bacteroidota; c_Bacteroidia; o_Bacteroidales; f_Bacteroidaceae; g_Prevotella; s_sp000434975 | 1.45 | 0.54 | 0.007 | 0.237 | Q2 |
| p_Firmicutes_A; c_Clostridia; o_Lachnospirales; f_Lachnospiraceae; g_Eubacterium_G; s_ventriosum | -1.39 | 0.54 | 0.010 | 0.237 | Q2 |
| p_Bacteroidota; c_Bacteroidia; o_Bacteroidales; f_Bacteroidaceae; g_Prevotella; s_stercorea | -2.22 | 0.82 | 0.007 | 0.237 | Q2 |
| p_Firmicutes_A; c_Clostridia; o_Lachnospirales; f_Lachnospiraceae; g_Coprococcus; s_sp000433075 | 0.97 | 0.37 | 0.010 | 0.237 | Q2 |
| p_Actinobacteriota; c_Actinomycetia; o_Actinomycetales; f_Bifidobacteriaceae; g_Bifidobacterium; s_adolescentis | -1.40 | 0.57 | 0.013 | 0.242 | Q2 |
| p_Bacteroidota; c_Bacteroidia; o_Bacteroidales; f_Bacteroidaceae; g_Phocaeicola; s_plebeius | 2.05 | 0.83 | 0.014 | 0.242 | Q2 |
|  |  |  |  |  |  |
| **Women** |  |  |  |  |  |
| p_Actinobacteriota; c_Actinomycetia; o_Actinomycetales; f_Bifidobacteriaceae; g_Bifidobacterium; s_bifidum | 3.13 | 0.82 | 0.000 | 0.025 | Q2 |
| p_Bacteroidota; c_Bacteroidia; o_Bacteroidales; f_Bacteroidaceae; g_Bacteroides; s_finegoldii | 2.33 | 0.73 | 0.002 | 0.119 | Q2 |
| p_Verrucomicrobiota; c_Verrucomicrobiae; o_Verrucomicrobiales; f_Akkermansiaceae; g_Akkermansia; s_muciniphila | 2.45 | 0.86 | 0.005 | 0.224 | Q2 |
| p_Bacteroidota; c_Bacteroidia; o_Bacteroidales; f_Rikenellaceae; g_Alistipes; s_onderdonkii | 1.86 | 0.68 | 0.006 | 0.236 | Q2 |
| The models were adjusted for age, body mass index (BMI), and days of vigorous physical activity for men and age and BMI for women. | | | | | |
| The highest quartile (Q4) group was set as the reference and compared with other groups. | | | | | |
| Only taxa with q < 0.25 are shown in the table. | | | | | |
| Abbreviations: log2FoldChange, log2 fold change estimate; lfcSE, standard error estimate for the log2 fold change estimate; p, *p*-value; q, adjusted *p*-value. | | | | | |

| **Table S2. Association of skeletal muscle index groups and taxa, stratified by age group** | | | | | |
| --- | --- | --- | --- | --- | --- |
| Taxonomy | log2FoldChange | lfcSE | p | q | value |
| **Men: age <45** |  |  |  |  |  |
| s__Ruminococcus_B gnavus | 3.31 | 1.09 | 0.003 | 0.063 | Q1 |
| s__Lachnospira sp900316325 | -2.78 | 0.95 | 0.004 | 0.063 | Q1 |
| s__Escherichia coli | 3.01 | 0.98 | 0.002 | 0.063 | Q1 |
| s__Bacteroides ovatus | 1.97 | 0.68 | 0.004 | 0.063 | Q1 |
| s__Lachnospira rogosae_A | -2.52 | 0.85 | 0.003 | 0.063 | Q1 |
| s__Phascolarctobacterium faecium | 2.63 | 0.89 | 0.003 | 0.063 | Q1 |
| s__Dysosmobacter sp001916835 | -2.52 | 0.84 | 0.003 | 0.063 | Q1 |
| s__Coprococcus sp900066115 | -2.39 | 0.73 | 0.001 | 0.063 | Q1 |
| s__Dialister sp000434475 | 1.13 | 0.32 | 0.000 | 0.063 | Q1 |
| s__Bacteroides fragilis | 2.86 | 1.07 | 0.008 | 0.097 | Q1 |
| s__CAG-127 sp900319515 | -2.42 | 0.91 | 0.008 | 0.097 | Q1 |
| s__Bifidobacterium longum | 2.01 | 0.77 | 0.009 | 0.103 | Q1 |
| s__Enterocloster sp001517625 | 2.83 | 1.14 | 0.014 | 0.130 | Q1 |
| s__Alloprevotella sp900540885 | 1.26 | 0.50 | 0.013 | 0.130 | Q1 |
| s__Faecalibacterium sp900539945 | -1.43 | 0.62 | 0.022 | 0.190 | Q1 |
| s__Dialister sp000434475 | 0.99 | 0.31 | 0.002 | 0.200 | Q2 |
| s__Blautia_A wexlerae | 0.99 | 0.44 | 0.026 | 0.217 | Q1 |
|  |  |  |  |  |  |
| **Men: age 45-64** |  |  |  |  |  |
| s__Escherichia coli | 3.51 | 0.90 | 0.000 | 0.017 | Q1 |
| s__Holdemanella porci | -2.22 | 0.61 | 0.000 | 0.048 | Q3 |
| s__Escherichia coli | 2.39 | 0.81 | 0.003 | 0.241 | Q3 |
|  |  |  |  |  |  |
| **Women: age <45** |  |  |  |  |  |
| - |  |  |  |  |  |
|  |  |  |  |  |  |
| **Women: age 45-64** |  |  |  |  |  |
| s__Alistipes onderdonkii | 5.11 | 1.08 | 0.000 | 0.001 | Q1 |
| s__Alistipes onderdonkii | 4.73 | 1.01 | 0.000 | 0.001 | Q2 |
| s__Mediterraneibacter torques | 3.94 | 1.18 | 0.001 | 0.084 | Q2 |
| s__Alloprevotella sp900539625 | -2.61 | 0.82 | 0.002 | 0.132 | Q1 |
| s__Bifidobacterium longum | 3.08 | 1.02 | 0.003 | 0.160 | Q2 |
| s__Prevotella copri_A | -3.38 | 1.13 | 0.003 | 0.174 | Q1 |
| s__Agathobacter rectalis | -2.68 | 1.00 | 0.008 | 0.193 | Q2 |
| s__Anaerostipes hadrus | 1.50 | 0.59 | 0.012 | 0.193 | Q2 |
| s__Prevotella copri | -4.22 | 1.66 | 0.012 | 0.193 | Q2 |
| s__AM51-8 sp003478275 | -2.94 | 1.08 | 0.007 | 0.193 | Q2 |
| s__Faecalibacterium sp900539945 | -1.65 | 0.64 | 0.011 | 0.193 | Q2 |
| s__Bifidobacterium bifidum | 2.93 | 1.16 | 0.012 | 0.193 | Q2 |
| s__Ruminococcus_D sp000434695 | 0.94 | 0.37 | 0.011 | 0.193 | Q2 |
| s__Bilophila wadsworthia | 1.82 | 0.73 | 0.014 | 0.199 | Q2 |
| s__Alloprevotella sp900539625 | -1.83 | 0.77 | 0.018 | 0.224 | Q2 |
| s__Lachnospira rogosae_A | -2.42 | 1.02 | 0.019 | 0.224 | Q2 |
| s__Bacteroides eggerthii | 1.85 | 0.79 | 0.022 | 0.231 | Q2 |
| s__CAG-353 sp900066885 | 1.05 | 0.45 | 0.022 | 0.231 | Q2 |
| The models were adjusted for age, body mass index (BMI), and days of vigorous physical activity for men and age and BMI for women. | | | | | |
| The highest quartile (Q4) group was set as the reference and compared with other groups. | | | | | |
| Only taxa with q < 0.25 are shown in the table. | | | | | |
| Abbreviations: log2FoldChange, log2 fold change estimate; lfcSE, standard error estimate for the log2 fold change estimate; p, *p*-value; q, adjusted *p*-value. | | | | | |

| **Table S3. Associations of skeletal muscle mass index quartiles and bacterial functional pathways** | | | | | |
| --- | --- | --- | --- | --- | --- |
| Pathway | log2FoldChange | lfcSE | *p* | q | value |
| **Men** | | | | | |
| MET-SAM-PWY: superpathway of S-adenosyl-L-methionine biosynthesis | 0.42 | 0.13 | 0.001 | 0.026 | Q3 |
| METSYN-PWY: superpathway of L-homoserine and L-methionine biosynthesis | 0.44 | 0.13 | 0.001 | 0.026 | Q3 |
| P441-PWY: superpathway of N-acetylneuraminate degradation | 0.44 | 0.13 | 0.001 | 0.026 | Q3 |
| PWY-5347: superpathway of L-methionine biosynthesis (transsulfuration) | 0.44 | 0.13 | 0.000 | 0.026 | Q3 |
| PWY-6270: isoprene biosynthesis I | 0.19 | 0.06 | 0.001 | 0.026 | Q3 |
| PWY66-409: superpathway of purine nucleotide salvage | 0.55 | 0.15 | 0.000 | 0.026 | Q3 |
| THISYN-PWY: superpathway of thiamine diphosphate biosynthesis I | 0.64 | 0.19 | 0.001 | 0.026 | Q3 |
| PWY-7560: methylerythritol phosphate pathway II | 0.19 | 0.06 | 0.002 | 0.046 | Q3 |
| ARG+POLYAMINE-SYN: superpathway of arginine and polyamine biosynthesis | 0.43 | 0.14 | 0.002 | 0.046 | Q3 |
| MET-SAM-PWY: superpathway of S-adenosyl-L-methionine biosynthesis | 0.42 | 0.14 | 0.003 | 0.060 | Q1 |
| METSYN-PWY: superpathway of L-homoserine and L-methionine biosynthesis | 0.44 | 0.15 | 0.003 | 0.060 | Q1 |
| P441-PWY: superpathway of N-acetylneuraminate degradation | 0.43 | 0.14 | 0.002 | 0.060 | Q1 |
| PWY-6270: isoprene biosynthesis I | 0.20 | 0.06 | 0.002 | 0.060 | Q1 |
| PWY-6353: purine nucleotides degradation II (aerobic) | 0.42 | 0.12 | 0.001 | 0.060 | Q1 |
| PWY-6608: guanosine nucleotides degradation III | 0.38 | 0.12 | 0.002 | 0.060 | Q1 |
| PWY66-409: superpathway of purine nucleotide salvage | 0.52 | 0.17 | 0.002 | 0.060 | Q1 |
| SER-GLYSYN-PWY: superpathway of L-serine and glycine biosynthesis I | 0.20 | 0.06 | 0.001 | 0.060 | Q1 |
| PWY-5347: superpathway of L-methionine biosynthesis (transsulfuration) | 0.41 | 0.14 | 0.003 | 0.063 | Q1 |
| SALVADEHYPOX-PWY: adenosine nucleotides degradation II | 0.43 | 0.15 | 0.004 | 0.073 | Q1 |
| P4-PWY: superpathway of L-lysine, L-threonine and L-methionine biosynthesis I | 0.53 | 0.19 | 0.006 | 0.090 | Q1 |
| PWY-5971: palmitate biosynthesis (type II fatty acid synthase) | 0.71 | 0.27 | 0.008 | 0.110 | Q3 |
| PWY-6353: purine nucleotides degradation II (aerobic) | 0.30 | 0.11 | 0.007 | 0.110 | Q3 |
| PWY-6608: guanosine nucleotides degradation III | 0.29 | 0.11 | 0.007 | 0.110 | Q3 |
| ARG+POLYAMINE-SYN: superpathway of arginine and polyamine biosynthesis | 0.41 | 0.16 | 0.008 | 0.111 | Q1 |
| PWY-7560: methylerythritol phosphate pathway II | 0.18 | 0.07 | 0.010 | 0.119 | Q1 |
| PWY0-781: aspartate superpathway | 0.48 | 0.19 | 0.010 | 0.119 | Q1 |
| THISYN-PWY: superpathway of thiamine diphosphate biosynthesis I | 0.54 | 0.21 | 0.010 | 0.119 | Q1 |
| COLANSYN-PWY: colanic acid building blocks biosynthesis | 0.17 | 0.07 | 0.011 | 0.147 | Q3 |
| GLCMANNANAUT-PWY: superpathway of N-acetylglucosamine, N-acetylmannosamine and N-acetylneuraminate degradation | 0.19 | 0.08 | 0.014 | 0.161 | Q3 |
| HEXITOLDEGSUPER-PWY: superpathway of hexitol degradation (bacteria) | 0.23 | 0.09 | 0.014 | 0.161 | Q3 |
| DAPLYSINESYN-PWY: L-lysine biosynthesis I | 0.35 | 0.14 | 0.016 | 0.169 | Q1 |
| PWY0-1479: tRNA processing | 0.35 | 0.15 | 0.019 | 0.188 | Q1 |
| ARG+POLYAMINE-SYN: superpathway of arginine and polyamine biosynthesis | 0.43 | 0.15 | 0.004 | 0.215 | Q2 |
| PWY-5971: palmitate biosynthesis (type II fatty acid synthase) | 0.87 | 0.28 | 0.002 | 0.215 | Q2 |
| THISYN-PWY: superpathway of thiamine diphosphate biosynthesis I | 0.60 | 0.20 | 0.003 | 0.215 | Q2 |
|  |  |  |  |  |  |
| **Women** |  |  |  |  |  |
| ANAEROFRUCAT-PWY: homolactic fermentation | 0.19 | 0.05 | 0.000 | 0.014 | Q2 |
| PENTOSE-P-PWY: pentose phosphate pathway | 0.30 | 0.08 | 0.000 | 0.014 | Q2 |
| PWY-5484: glycolysis II (from fructose 6-phosphate) | 0.31 | 0.08 | 0.000 | 0.014 | Q2 |
| GLYCOLYSIS: glycolysis I (from glucose 6-phosphate) | 0.29 | 0.08 | 0.000 | 0.014 | Q2 |
| PWY-6901: superpathway of glucose and xylose degradation | 0.27 | 0.08 | 0.000 | 0.014 | Q2 |
| ANAGLYCOLYSIS-PWY: glycolysis III (from glucose) | 0.14 | 0.05 | 0.004 | 0.050 | Q2 |
| BIOTIN-BIOSYNTHESIS-PWY: biotin biosynthesis I | 0.47 | 0.16 | 0.004 | 0.050 | Q2 |
| DAPLYSINESYN-PWY: L-lysine biosynthesis I | 0.44 | 0.16 | 0.005 | 0.050 | Q2 |
| FASYN-ELONG-PWY: fatty acid elongation -- saturated | 0.53 | 0.18 | 0.003 | 0.050 | Q2 |
| FUC-RHAMCAT-PWY: superpathway of fucose and rhamnose degradation | 0.47 | 0.16 | 0.003 | 0.050 | Q2 |
| GLYCOLYSIS-E-D: superpathway of glycolysis and the Entner-Doudoroff pathway | 0.24 | 0.08 | 0.005 | 0.050 | Q2 |
| PWY-4041: gamma-glutamyl cycle | 0.47 | 0.15 | 0.002 | 0.050 | Q2 |
| PWY-5989: stearate biosynthesis II (bacteria and plants) | 0.52 | 0.18 | 0.005 | 0.050 | Q2 |
| PWY-6282: palmitoleate biosynthesis I (from (5Z)-dodec-5-enoate) | 0.55 | 0.19 | 0.004 | 0.050 | Q2 |
| PWY-6519: 8-amino-7-oxononanoate biosynthesis I | 0.50 | 0.17 | 0.004 | 0.050 | Q2 |
| PWY-7388: octanoyl-[acyl-carrier protein] biosynthesis (mitochondria, yeast) | 0.57 | 0.19 | 0.003 | 0.050 | Q2 |
| PWY-7664: oleate biosynthesis IV (anaerobic) | 0.54 | 0.18 | 0.003 | 0.050 | Q2 |
| PWY0-862: (5Z)-dodecenoate biosynthesis I | 0.55 | 0.19 | 0.003 | 0.050 | Q2 |
| HOMOSER-METSYN-PWY | 0.49 | 0.18 | 0.007 | 0.052 | Q2 |
| MET-SAM-PWY: superpathway of S-adenosyl-L-methionine biosynthesis | 0.42 | 0.16 | 0.007 | 0.052 | Q2 |
| METSYN-PWY: superpathway of L-homoserine and L-methionine biosynthesis | 0.44 | 0.16 | 0.007 | 0.052 | Q2 |
| P4-PWY: superpathway of L-lysine, L-threonine and L-methionine biosynthesis I | 0.50 | 0.18 | 0.007 | 0.052 | Q2 |
| PWY-5347: superpathway of L-methionine biosynthesis (transsulfuration) | 0.42 | 0.15 | 0.006 | 0.052 | Q2 |
| PWY-5659: GDP-mannose biosynthesis | 0.24 | 0.09 | 0.007 | 0.052 | Q2 |
| SER-GLYSYN-PWY: superpathway of L-serine and glycine biosynthesis I | 0.16 | 0.06 | 0.007 | 0.052 | Q2 |
| PWY0-781: aspartate superpathway | 0.47 | 0.18 | 0.009 | 0.064 | Q2 |
| P441-PWY: superpathway of N-acetylneuraminate degradation | 0.44 | 0.17 | 0.011 | 0.073 | Q2 |
| GLCMANNANAUT-PWY: superpathway of N-acetylglucosamine, N-acetylmannosamine and N-acetylneuraminate degradation | 0.22 | 0.09 | 0.012 | 0.074 | Q2 |
| NAGLIPASYN-PWY: lipid IVA biosynthesis (E. coli) | 0.44 | 0.18 | 0.012 | 0.074 | Q2 |
| POLYISOPRENSYN-PWY: polyisoprenoid biosynthesis (E. coli) | 0.32 | 0.13 | 0.013 | 0.074 | Q2 |
| PWY0-845: superpathway of pyridoxal 5'-phosphate biosynthesis and salvage | 0.38 | 0.15 | 0.013 | 0.074 | Q2 |
| TCA: TCA cycle I (prokaryotic) | 0.25 | 0.10 | 0.013 | 0.074 | Q2 |
| FERMENTATION-PWY: mixed acid fermentation | 0.51 | 0.22 | 0.019 | 0.100 | Q2 |
| PWY-7282: 4-amino-2-methyl-5-diphosphomethylpyrimidine biosynthesis II | 0.25 | 0.11 | 0.019 | 0.100 | Q2 |
| COLANSYN-PWY: colanic acid building blocks biosynthesis | 0.14 | 0.06 | 0.020 | 0.101 | Q2 |
| PWY-7117: C4 photosynthetic carbon assimilation cycle, PEPCK type | 0.56 | 0.25 | 0.023 | 0.110 | Q2 |
| PYRIDOXSYN-PWY: pyridoxal 5'-phosphate biosynthesis I | 0.37 | 0.16 | 0.023 | 0.110 | Q2 |
| PWY-241: C4 photosynthetic carbon assimilation cycle, NADP-ME type | 0.55 | 0.25 | 0.025 | 0.118 | Q2 |
| HEXITOLDEGSUPER-PWY: superpathway of hexitol degradation (bacteria) | 0.32 | 0.14 | 0.027 | 0.122 | Q2 |
| PWY-5913: partial TCA cycle (obligate autotrophs) | 0.59 | 0.27 | 0.029 | 0.128 | Q2 |
| GLUCONEO-PWY: gluconeogenesis I | 0.17 | 0.08 | 0.031 | 0.133 | Q2 |
| PWY-6936: seleno-amino acid biosynthesis (plants) | 0.23 | 0.11 | 0.031 | 0.133 | Q2 |
| NONOXIPENT-PWY: pentose phosphate pathway (non-oxidative branch) I | 0.18 | 0.08 | 0.037 | 0.151 | Q2 |
| PWY-5188: tetrapyrrole biosynthesis I (from glutamate) | 0.22 | 0.11 | 0.038 | 0.154 | Q2 |
| ANAEROFRUCAT-PWY: homolactic fermentation | 0.17 | 0.06 | 0.004 | 0.173 | Q1 |
| HOMOSER-METSYN-PWY: L-methionine biosynthesis I | 0.58 | 0.20 | 0.005 | 0.173 | Q1 |
| MET-SAM-PWY: superpathway of S-adenosyl-L-methionine biosynthesis | 0.51 | 0.17 | 0.003 | 0.173 | Q1 |
| METSYN-PWY: superpathway of L-homoserine and L-methionine biosynthesis | 0.53 | 0.18 | 0.004 | 0.173 | Q1 |
| PWY-5347: superpathway of L-methionine biosynthesis (transsulfuration) | 0.50 | 0.17 | 0.004 | 0.173 | Q1 |
| FUC-RHAMCAT-PWY: superpathway of fucose and rhamnose degradation | 0.48 | 0.18 | 0.007 | 0.187 | Q1 |
| PWY-5484: glycolysis II (from fructose 6-phosphate) | 0.25 | 0.09 | 0.007 | 0.187 | Q1 |
| GLYCOGENSYNTH-PWY: glycogen biosynthesis I (from ADP-D-Glucose) | 0.15 | 0.08 | 0.048 | 0.190 | Q2 |
| GLYCOLYSIS: glycolysis I (from glucose 6-phosphate) | 0.23 | 0.09 | 0.010 | 0.229 | Q1 |
| PWY-2941: L-lysine biosynthesis II | 0.26 | 0.14 | 0.062 | 0.240 | Q2 |
| The models were adjusted for age, body mass index (BMI), and days of vigorous physical activity for men and age and BMI for women. | | | | | |
| The highest quartile (Q4) group was set as the reference and compared with other groups. | | | | | |
| Only pathways with q < 0.25 are shown. | | | | | |
| Abbreviations: log2FoldChange, log2 fold change estimate; lfcSE, standard error estimate for the log2 fold change estimate; p, p-value; q, adjusted p-value. | | | | | |

| **Table S4. Associations of skeletal muscle mass index groups and bacterial metabolite in men** | | | | | |
| --- | --- | --- | --- | --- | --- |
| Metabolite | log2FoldChange | lfcSE | p | q | value |
| N-oleoylethanolamine | 0.16 | 0.05 | 0.001 | 0.072 | Q2 |
| deoxycholic acid | -0.16 | 0.05 | 0.002 | 0.096 | Q1 |
| linoleoyl ethanolamide | 0.08 | 0.03 | 0.005 | 0.096 | Q1 |
| N-oleoylethanolamine | 0.15 | 0.05 | 0.004 | 0.096 | Q1 |
| cholesterol | 0.11 | 0.04 | 0.003 | 0.096 | Q1 |
| pseudouridine | -0.05 | 0.02 | 0.009 | 0.130 | Q1 |
| chenodeoxycholate deoxycholate | -0.09 | 0.04 | 0.02 | 0.243 | Q1 |
| The models were adjusted for age, body mass index (BMI), and days of vigorous physical activity. | | | | | |
| The highest quartile (Q4) group was set as the reference and compared with others. | | | | | |
| Only metabolites with q <0.25 are shown. | | | | | |
| Abbreviations: log2FoldChange, log2 fold change estimate; lfcSE, standard error estimate for the log2 fold change estimate; p, p value; q, adjusted p-value. | | | | | |
